# Innate immune activation: Parallels in alcohol use disorder and Alzheimer’s disease

**DOI:** 10.3389/fnmol.2022.910298

**Published:** 2022-09-09

**Authors:** Adriana Ramos, Radhika S. Joshi, Gyongyi Szabo

**Affiliations:** ^1^Beth Israel Deaconess Medical Center, Harvard Medical School, Boston, MA, United States; ^2^Broad Institute of MIT and Harvard, Cambridge, MA, United States

**Keywords:** alcohol, microglia, Alzheimer’s disease, innate immunity, liver-brain axis, neuroinflammation

## Abstract

Alcohol use disorder is associated with systemic inflammation and organ dysfunction especially in the liver and the brain. For more than a decade, studies have highlighted alcohol abuse-mediated impairment of brain function and acceleration of neurodegeneration through inflammatory mechanisms that directly involve innate immune cells. Furthermore, recent studies indicate overlapping genetic risk factors between alcohol use and neurodegenerative disorders, specifically regarding the role of innate immunity in the pathomechanisms of both areas. Considering the pressing need for a better understanding of the relevance of alcohol abuse in dementia progression, here we summarize the molecular mechanisms of neuroinflammation observed in alcohol abuse and Alzheimer’s disease, the most common cause of dementia. In addition, we highlight mechanisms that are already established in the field of Alzheimer’s disease that may be relevant to explore in alcoholism to better understand alcohol mediated neurodegeneration and dementia, including the relevance of the liver-brain axis.

## Introduction

Alcohol use disorder (AUD), characterized by uncontrolled alcohol drinking, is one of the leading causes of preventable deaths in the United States with a significant socio-economic burden on society (Spillane et al., 2020). Alcoholism is associated with dysfunction of multiple organs leading to cirrhosis, cardiovascular complications, and neuronal dysfunction. Along with the acute sedative effect of ethanol, chronic alcohol abuse is associated with cognitive dysfunction, dementia, neurodegeneration and gray and white matter shrinkage (Crews, 2008; Wilcox et al., 2014; Zahr, 2014; Yang et al., 2016; Schwarzinger et al., 2018; Rehm et al., 2019).

Over the past few decades, we and others have shown that alcohol consumption leads to excessive inflammation in vital organs including liver, intestines, and brain (Crews, 2012; Szabo et al., 2012; Szabo and Lippai, 2014; Bishehsari et al., 2017). Ethanol-induced innate immune activation in the liver plays a central role in alcoholic liver disease and several strategies of intervention that involve targeting the peripheral innate immune system have been proposed (Xu et al., 2017; Tornai and Szabo, 2020). Moreover, alcohol-induced innate immune activation in the central nervous system (CNS) has been shown to mediate neurotoxicity and ethanol-induced behaviors including alcohol addiction and cognitive decline in preclinical and clinical settings (Crews, 2008; Henriques et al., 2018; Erickson et al., 2019b) (see also Tables 1, 2). Indeed, antagonism of IL-1 receptor signaling and the NLRP3 inflammasome reduces inflammation and alcohol consumption (Marshall et al., 2016; Erickson et al., 2019b; Lowe et al., 2020a). In addition, genetic studies demonstrated involvement of the innate immune system in the pathogenesis of AUD. These studies reported polymorphisms associated with AUD in immune-related genes such as NFκB or IL-10 (Crews, 2012; Kapoor et al., 2021). Furthermore, the integration of GWAS and eQTL data helped identify causal variants for AUD involved in the regulation of PU.1, an important transcription factor for myeloid cells and a demonstrated master regulator of the inflammatory response in microglia (Kapoor et al., 2021; Pimenova et al., 2021).

**TABLE 1:** Features of innate immune activation in rodent models of alcohol use disorder.

	Outcome compared to control	Technique	Model	Ethanol paradigm	Brain region	References
Microglia proliferation	↑ proliferation	Cx3cr1-EYFP positive cells/IF	Mouse	10d binge	Prefrontal cortex	Socodato et al., 2020
		Iba1 positive cells/IF		5m chronic treatment	Motor cortex	Alfonso-Loeches et al., 2016
		Iba1 positive cells/IF		10d binge	Cortex	Qin and Crews, 2012a
		Cd11b^+^ CD45^low^ counts/FC	Rat	4d binge	Hippocampus, Entorhinal cortex	Peng et al., 2017
		Iba1 positive cells/IF		4d binge	Hippocampus	Marshall et al., 2013
Microglia morphology	Hyper-ramified	Iba1 positive cells/IF	Mouse	10d binge	Prefrontal cortex	Socodato et al., 2020
	Bushy	Iba1 positive cells/IF		6weeks LDC	Hippocampus	Lowe et al., 2020b
		Iba1 positive cells/DAB		NIAAA	Hippocampus, Cortex	Lowe et al., 2018
		Iba1 positive cells/DAB		5m chronic treatment	Motor cortex	Alfonso-Loeches et al., 2016
		Cd11b (OX-42) positive cells/IHC	Rat	4d binge	Hippocampus	Marshall et al., 2013
Microglia lysosomal expression and phagocytosis	↑ phagocytosis ↑ CD68 expression	Iba1, CD68, PSD-95/IF	Mouse	10d binge	Cortex	Socodato et al., 2020
	↑ phagocytosis of beads	IF	Mouse primary microglia	70 mM Ethanol, 90 min	Cortex	
	↑ CD68	qRT- PCR	Mouse	Single binge	Total brain	Walter and Crews, 2017
		qRT- PCR, WB		5m chronic treatment	Cortex	Alfonso-Loeches et al., 2016
	= CD68	CD68/IHC	Rat	4d binge	Hippocampus, entorhinal cortex	Marshall et al., 2013
	↓ CD68	CD68/FC, qRT-PCR	Mouse	6week LDC	Hippocampus	Lowe et al., 2020b
	↓ phagocytosis of Aβ	FC	Rat primary microglia	75 mM Ethanol, 24 h	Frontal cortex	Kalinin et al., 2018
Cytokine and chemokine expression	↑ TNFα, IL-1β	ELISA	Mouse	6wk LDC	Hippocampus	Lowe et al., 2020b
	↑ TNFα, ↓ IL-6, CCL2	qRT-PCR		10d binge	Cortex	Socodato et al., 2020
	↑ TNFα, IL-18, MCP-1, IL-17, IL-23	qRT-PCR		NIAAA	Cortex	Lowe et al., 2018
	↑ TNFα, IL-6, MCP-1	ELISA		10d binge	Total brain	Qin et al., 2008; Qin and Crews, 2012a
	↑ IFN-γ, IL-33, Cx3CL1, CXCL2, ↓CCL4	ELISA, qRT-PCR		5m chronic treatment	Cortex	Alfonso-Loeches et al., 2016
	↑TNFα, IL-1β, IL-17, IFN-γ, MCP-1, MIP-1, CX3CL1	ELISA		5m chronic treatment	Striatum	Pascual et al., 2015
	↑ TNFα, IL-1β, IL-6, CCL2, 1L-10, 1L-4	qRT-PCR		Single binge	Total brain	Walter and Crews, 2017
	↑ TNFα, CCL2	qRT-PCR	BV2 cell line	85 mM Ethanol, 24 h	N/A	
	↑ TNFα, IL-1β	ELISA, qRT-PCR	Mouse and Rat primary microglia	10–100 mM Ethanol, 3-24 h	Cortex	Fernandez-Lizarbe et al., 2009
	↑ TNFα, IL-1β	qRT-PCR	Rat organotypic slice cultures	100 mM Ethanol, 24–96 h	Hippocampal-Entorhinal cortex	Crews et al., 2021
	= TNFα, IL-6 (Trend toward ↓)	ELISA	Rat	4d binge	Hippocampus, Entorhinal cortex	Marshall et al., 2013
Expression of immune mediators	↑ Acetylated HMGB1	WB	Mouse	6wk LDC	Cerebellum	Lippai et al., 2013b
	↑ HMGB1	qRT-PCR		NIAAA	Cortex	Lowe et al., 2018
	↑ iNOS, COX-2	qRT-PCR		Single binge	Total brain	Walter and Crews, 2017
	↑ HMGB1 in EVs	ELISA	Rat organotypic slice cultures	25–100 mM Ethanol, 48 h	Hippocampus-Entorhinal cortex	Coleman et al., 2017
	↑ iNOS, COX-2	WB	Mouse and Rat primary microglia	50 mM Ethanol up to 24 h	Cortex	Fernandez-Lizarbe et al., 2009

4d binge: 4 day alcohol binge model, ∼ 9 g/kg/day in rats.

10d binge: 10 daily alcohol binges- 1.5 g/kg or 5 g/kg in mice.

5m chronic treatment: 5 month chronic alcohol administration through drinking water at 10% v/v in mice.

6wk LDC- 6 weeks of Lieber-DeCarli liquid diet with 5% v/v ethanol, in mice.

NIAAA: 10 days of Lieber-DeCarli liquid diet with 5% v/v ethanol + 5g/kg alcohol binge 9h before sacrifice in mice.

Primary cultures: Primary microglia cultures derived from either rat or mouse.

Single binge: 6g/kg alcohol binge.

DAB- 3,3’Diaminobenzidine.

IF- Immunofluorescence.

IHC-Immunohistochemistry.

FC- Flow Cytometry.

**TABLE 2:** Mechanisms that lead to ethanol-reactive microglia in alcohol use disorder.

	Brief description	Model	Ethanol paradigm	Brain region	References
**TLR4 activation**					
Features of pathway activation	↑ TLR4, Localization of TLR4 in lipid rafts	Mouse & Rat primary microglia	50 mM Ethanol up to 24 h	Cortex	Fernandez-Lizarbe et al., 2013
	↑ MAPK, JNK signaling	Mouse and Rat primary microglia	10–100 mM Ethanol, 3–24 h	Cortex	Fernandez-Lizarbe et al., 2009
	↑ Release of TNFα				
Phenotype in TLR4 KO	**Protection against ethanol-induced:**	Mouse	5m chronic treatment	Cortex	Alfonso-Loeches et al., 2016
	Alterations in microglia morphology, proliferation				
	Peripheral macrophage infiltration				
	Increased ROS and BBB damage				
	Increase in NLRP3				
	Cytokine, chemokine expression (IL-1β, TLR4, IL-33, CXCL2, CX3CL1, IFN-γ)				
	Cytokine, chemokine expression (TNFα, IL-1β, IFN-γ, IL-17, MCP-1, MIP-1)			Striatum	Pascual et al., 2015
	Anxiety				
**Inflammasome activation**					
Features of pathway activation	↑ Cleaved IL-1β, Caspase-1	Mouse	6wk LDC	Cerebellum	Lippai et al., 2013b
	↑ NLRP3, Cleaved Caspase-1, ASC dimerization, IL-1β release	Mouse Primary cultures	10–50 mM Ethanol	Cortex	Alfonso-Loeches et al., 2016
	↑Caspase-1 immunoreactivity	Mouse	5m chronic treatment	Cortex	Alfonso-Loeches et al., 2016
Phenotypes on inflammasome inhibition	Protection against cytokine, chemokine expression (TNFα, IL-1β, MCP-1)		6wk LDC	Cerebellum	Lippai et al., 2013b
	Reduction in alcohol consumption and ethanol preference		Two-bottle choice test	N/A	Lowe et al., 2020a
**Activation of Complement system**					
Features of pathway activation	↑ *C1q*	Slice cultures	100 mM Ethanol, 24–96 h	Hippocampal-Entorhinal cultures	Crews et al., 2021
	↑ *C1q*	Mouse	6wk LDC	Hippocampus, Cerebellum	Lowe et al., 2020b
	↑ *C1q*, *C3*, *C3ar1*	Rat primary cultures	75 mM Ethanol, 24 h	Frontal cortex	Kalinin et al., 2018
	↓ *C1q*	Mouse	10d binge	Cortex	Socodato et al., 2020

Risk alleles for Alzheimer’s disease (AD) are also enriched in myeloid cells and overlapping variants for AUD and AD exist in enhancer regions of the *SPI1* gene (gene that encodes for PU.1 protein) (Bertram et al., 2008; Guerreiro et al., 2013). In addition to genetics, AUD and AD share common pathophysiological mechanisms involving important neuroinflammation and neurodegeneration hallmarks (see section “Alcohol abuse and progression of AD”) (Bertram et al., 2008; Crews, 2008; Guerreiro et al., 2013; Heneka et al., 2013; Hong et al., 2016; Hammond et al., 2019; Kapoor et al., 2021). Over the years, several features of microglia (including morphology, motility, proliferation, and secretion) have been studied in detail in various models of AD and AUD independently and are reviewed elsewhere. In this review, we discuss the common pathways that lead to reactive microglia in AD and AUD, and introduce mechanisms explored in AD that may be relevant in the context of AUD. Furthermore, we discuss the implication of these common inflammatory mechanisms on the potentially detrimental effect of alcohol abuse on the progression of AD.

With these components in mind, this review focuses on the role of innate immunity in ethanol-induced neuro-inflammation. Since microglia are the primary mediators of innate immunity in the central nervous system, we describe features of microglia activation and their mechanism in AUD.

We also discuss the contribution of the peripheral innate immune system in regulating brain function, especially in the context of AUD. Since the liver is the primary site of alcohol metabolism and a major regulator of innate-immune activation, we hypothesize that, in addition to the direct effect of alcohol or its metabolites on the brain, liver-derived peripheral cytokines, hepatotoxins and circulating immune populations can communicate to the CNS to induce inflammation in AUD. Finally, since these pathways may provide novel insight into the role of alcohol abuse on features of AD, we discuss myeloid cell infiltration and the role of liver-brain axis in AD.

Hence, this review is divided into three broad sections that describe (a) innate immunity in AUD, (b) innate immunity in AD, and (c) studies where effects of alcohol abuse on features of AD have been investigated. This comparison between the features of innate immune activation in AUD and AD will provide new opportunities for future investigations, especially in the context of the dual insult models of AUD and AD. A list of abbreviations and gene symbols is included (see Table 3).

**TABLE 3:** Abbreviations and gene symbols.

AD- Alzheimer’s Disease	LPS-Lipopolysaccharides	CCR-C-C Motif Chemokine Receptor	C3- Complement Component 3	BACE1- Beta-Secretase 1
AUD- Alcohol Use Disorder	TNFα- Tumor Necrosis Factor α	CCL-2- C-C Motif Chemokine Ligand 2	TGFβ-Transforming Growth Factor β	GWAS – Genome-Wide Association Study
EAE-Experimental Autoimmune Encephalomyelitis	DAMPs- Damage-Associated Molecular Patterns	CX3CR1- C-X3-C Motif Chemokine Receptor 1	APP-Amyloid β Precursor Protein	PU.1-Purine rich transcription factor, encoded by *SPI-1* (Spi-1 Proto-Oncogene)
TBI-Traumatic Brain Injury	HMGB1- High-Mobility Group protein 1	CXCL-1- Chemokine (C-X-C motif) ligand 1	BBB- Blood Brain Barrier	TREM2-Triggering Receptor Expressed On Myeloid Cells 2
PRRs- Pattern Recognition Receptors	iNOS- Nitric Oxide Synthases, inducible isoform	MIP-1- Macrophage Inflammatory Proteins	ALS- Amyotrophic Lateral Sclerosis	mir-155- MicroRNA 155
TLRs- Toll Like Receptors	COX-2- Cyclooxygenase-2	MCP-1- Monocyte Chemoattractant Protein-1	FTD- Frontotemporal Dementia	MAPT-Microtubule Associated Protein Tau
NLRP3- NLR Family Pyrin Domain Containing 3	IL- Interleukin family	IFN-Interferon	KO- Knockout Out (mice)	ROS- Reactive Oxygen Species
ASC- Apoptosis-associated Speck-like protein containing a CARD	IL-1Ra- Interleukin-1 Receptor Antagonist	Let-7b – *Lethal-7b*	LRP-1- LDL Receptor Related Protein 1	CNS- Central Nervous System
ASH- Alcoholic Steatohepatitis	NASH- Non-Alcoholic Steatohepatitis	NF-kB – Nuclear Factor kappa B	eQTL – Expression Quantitative Trait Loci	

## Brain innate immunity in alcohol use disorder

### Neuroinflammatory mechanisms mediated by microglia

#### Microglia proliferation and morphology in alcohol use disorder

**FIGURE 1:**
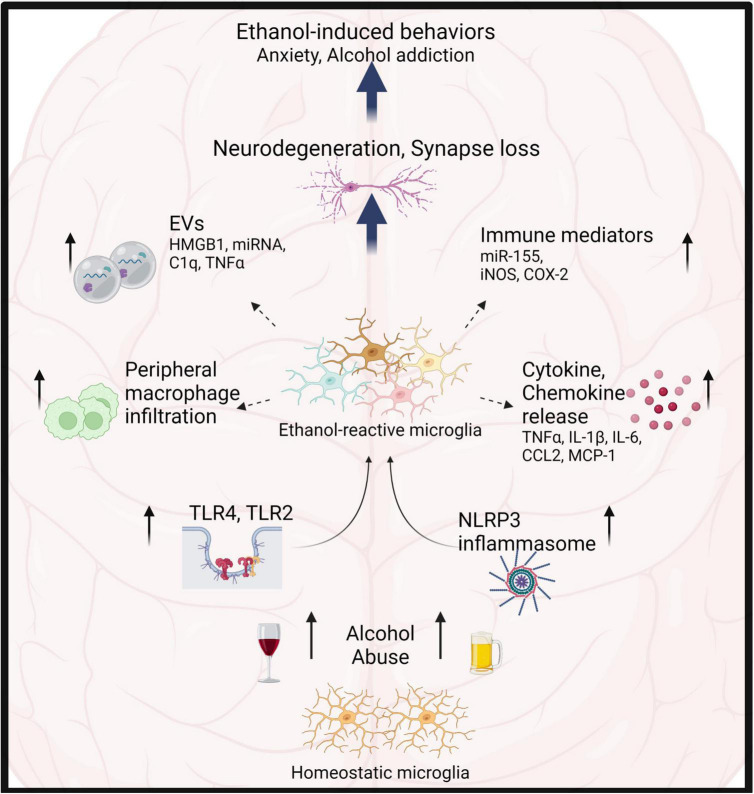
Microglia play critical role in alcohol-induced neuroinflammation. Alcohol abuse leads to microglia activation primarily through TLR4, 2, and NLRP3 inflammasomes. Consequently, microglia show increased proliferation, morphological transformation, release of cytokines, chemokines, EVs and immune mediators. This results in infiltration of peripheral macrophages, neurotoxicity, synapse loss and modulation of ethanol-induced behaviors.

Effects of alcohol are wide spread in the brain and include cytokine and chemokine release, immune cell infiltration, synapse loss and neuronal death (Figure 1 and Table 1). Microglia, the resident immune cells of the brain, are the key mediators in these processes. Under physiological conditions, microglia survey the brain parenchyma (Nimmerjahn et al., 2005) and take care of many critical CNS functions, enabled by their capacity to release soluble factors, engulf extracellular material and dead cells or migrate to the sites of infection (Butovsky et al., 2014; Paolicelli et al., 2022). Transcriptomic studies demonstrate that the pattern of microglia states varies with the etiology of the disease and severity though commonalities have been found and highlighted across diseases including AUD (Masuda et al., 2020; Chen and Colonna, 2021). Microglia can respond to inflammation through changes in cell morphology, motility, cytokine release, transcriptomic and epigenetic changes. These responses and thus the reactive state of microglia varies with the stimulus and its intensity (Paolicelli et al., 2022). Traditionally, studies have tried to classify microglia as homeostatic Vs activated or M1 (pro-inflammatory) Vs M2 (alternatively activated, anti-inflammatory) based on (1) Morphological features (2) Surface antigen markers (3) Phagocytic ability (4) Cytokine release (Beynon and Walker, 2012; Marshall et al., 2013; Crews and Vetreno, 2016; Paolicelli et al., 2022). In this regard, homeostatic microglia have a ramified branched appearance. In response to mild inflammatory stimuli, microglia can show increased branching and a hyper-ramified appearance, also may express anti-inflammatory markers (also referred as M2 markers). During a more severe inflammatory condition, microglia may appear bushy with fewer, thicker branches and bigger soma and secrete pro-inflammatory cytokines, resembling M1 state. Finally, under extreme inflammatory conditions microglia appear amoeboid (Frautschy et al., 1998; Beynon and Walker, 2012). However, recent advances challenge the direct correlation between microglia morphology and their reactiveness and phagocytic ability (Paolicelli et al., 2022). Thus this simplistic classification is now considered out of date (Paolicelli et al., 2022). Therefore, throughout this review, we will refrain from classifying microglia in either of these categories and instead describe various features as reported in literature.

In response to alcohol, microglia can undergo proliferation and also exhibit a spectrum of morphological and molecular transformations, changing their overall function. Indeed, increased microglia proliferation has been reported in the cortex and hippocampus of chronic ethanol-treated mice (McClain et al., 2011; Qin and Crews, 2012a; Marshall et al., 2013; Alfonso-Loeches et al., 2016; Peng et al., 2017; Socodato et al., 2020) and post-mortem brain samples of AUD patients (Qin et al., 2008; Rubio-Araiz et al., 2017).

Several preclinical models of AUD report a moderate change in microglia morphology toward more bushy appearance (Figure 1 and Table 1) (Fernandez-Lizarbe et al., 2009; Lowe et al., 2020b; Socodato et al., 2020).

#### Microglia phagocytosis in alcohol use disorder

Microglia are the resident phagocytes of the brain and are involved in the clearance of non-functional synapses, pathogenic peptides, myelin debris, and dying neurons (Wyss-Coray et al., 2001; Takahashi et al., 2005; Schafer et al., 2012; Lampron et al., 2015). Microglia exhibit increased phagocytosis during development and in response to inflammatory stimuli (Paolicelli et al., 2011; Kettenmann et al., 2013). The effect of ethanol on microglia phagocytosis appears to be very dynamic and context dependent (Table 1). Walter and Crews observed that the levels of lysosomal marker CD68 changes over the withdrawal period of 24 h after acute alcohol binge, with an initial decline followed by a significant increase that peaks 18 h post binge (Walter and Crews, 2017). Chronic alcohol treatment also appears to increase CD68 levels in the cortex and cerebellum (Lippai et al., 2013a; Alfonso-Loeches et al., 2016; Socodato et al., 2020), although Lowe et al. reported a reduction in CD68 levels in the hippocampi (Lowe et al., 2020b). Furthermore, ethanol pre-treatment reduces phagocytosis of Aβ in primary microglia cultures (Kalinin et al., 2018), consistent with the effect of alcohol on peripheral macrophages (Bukong et al., 2018). Thus, duration of ethanol withdrawal, brain region of interest and phagocytic substrate are important in the assessment of ethanol’s effect on microglia phagocytosis (Table 1).

#### Release of cytokines and DAMPs by microglia in alcohol use disorder

As mentioned above, alcohol-reactive microglia secrete inflammatory cytokines and chemokines, which are detected at the tissue level in the brain parenchyma or cell culture supernatants (Figure 1 and Table 1). Numerous studies have reported ethanol-induced increases in cytokines and chemokines (TNFα, IL-1β, IL-6, IL-17, IL-23, CCL2, CXCL1, MIP-1α) in various brain regions (Qin et al., 2008; Alfonso-Loeches et al., 2010; Qin and Crews, 2012a; Lippai et al., 2013a,b; Pascual et al., 2015; Walter and Crews, 2017; Lowe et al., 2018, 2020b; Xu et al., 2020). Furthermore, *in vitro* experiments show that direct stimulation of microglia with ethanol produces TNFα, IL-1β and immune mediators such as iNOS, COX-2, among other cytokines (Fernandez-Lizarbe et al., 2009; Walter and Crews, 2017; Lee et al., 2021). Microglia depletion experiments (see also- Section “Insights from microglia depletion experiments in AUD”) highlight microglia as the major source of cytokine secretion in AUD. In addition some studies have reported increase in anti-inflammatory IL-4 and IL-10 in brain during ethanol withdrawal, though direct contribution of microglia was demonstrated to be minimal (Marshall et al., 2013; Walter and Crews, 2017). Interestingly, ethanol increased both pro-inflammatory (CD86, CD32) and anti-inflammatory surface markers (CD206) on microglia (Peng and Nixon, 2021). Thus, M1-M2 classification, abundantly used in the alcohol field to describe the dichotomy of pro-inflammatory Vs anti-inflammatory states of microglia are insufficient to bring out nuances of reactive states in AUD (see also- Alzheimer’s disease-related mechanisms unexplored in alcoholism: disease associated microglia).

In addition, chronic ethanol treatment up-regulates expression of DAMPs such as HMGB1 in pre-clinical models and post-mortem alcoholic patient samples (Crews et al., 2013; Lippai et al., 2013b; Coleman et al., 2017, 2018). Interestingly, HMGB-1 and micro-RNA let-7b complexes are released in micro-vesicles upon ethanol stimulation and mediate neuronal cell death through TLR-7 (Coleman et al., 2017). Moreover, HMGB1 forms complexes with IL-1β in hippocampal tissue of alcoholic patients and these complexes are shown to have enhanced inflammatory properties (Coleman et al., 2018).

#### Release of extracellular vesicles by microglia in alcohol use disorder

**FIGURE 2:**
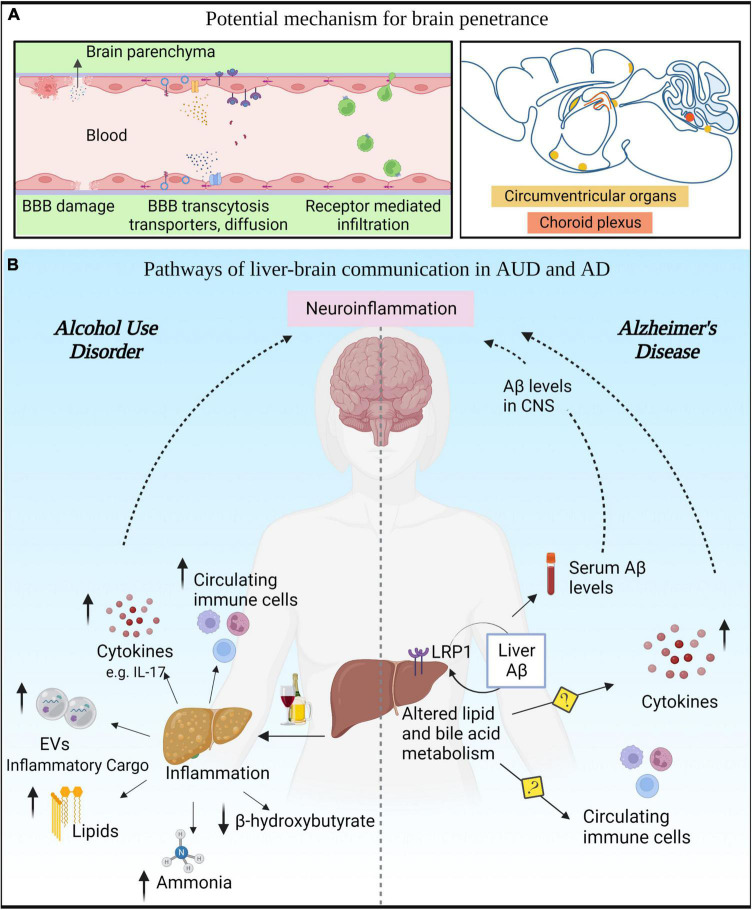
Liver-brain axis in alcohol use disorder (AUD) and Alzheimer’s disease (AD). **(A)** Various modes of communication between the peripheral circulation and the brain. **(B)** AUD leads to innate immune activation in liver that results in secretion of cytokines, chemokines, toxic lipids, EVs, and hepatotoxins in the circulation that can reach the CNS. In AD, altered liver metabolism of lipids and bile acids can modulate Aβ levels in the periphery and consequently in the CNS.

Extracellular vesicles (EVs) are membrane bound vesicles released by the endocytic machinery into the extracellular space by a variety of cell types including microglia. EVs carry biomolecules such as nucleic acids, peptides, and lipids depending on the cell type of origin and stimulus (Thery et al., 2002; Gupta and Pulliam, 2014; Yang et al., 2018). Circulating EVs can be stable and cross the blood brain barrier, forming a potential route of liver-brain communication and therapeutic intervention (Gupta and Pulliam, 2014) (Figure 2). Microglia have been shown to release EVs in response to ATP (Bianco et al., 2005) and inflammatory stimuli such as LPS (Verderio et al., 2012; Yang et al., 2018). In addition, microglia-derived EVs are thought to propagate inflammation in mouse models of EAE and TBI (Verderio et al., 2012; Kumar et al., 2017). Importantly, microglia-derived EVs contribute toward processing, clearance and spread of Aβ and tau pathology (Rajendran et al., 2006; Tamboli et al., 2010; Asai et al., 2015; Polanco et al., 2016). We and others have shown that ethanol stimulates release of exosomes in plasma that contribute to the pathogenesis of alcoholic hepatitis (Momen-Heravi et al., 2015; Saha et al., 2016; Ibanez et al., 2020; Babuta and Szabo, 2022). In addition, ethanol treatment increases pro-inflammatory cargo of microglia derived EVs including micro-RNA let-7b, C1q, *Tnfa* mRNA, and HMGB1 (Coleman et al., 2017; Mukherjee et al., 2020; Crews et al., 2021) (Figure 1). Indeed, Crews et al. showed that ethanol-induced EVs alone are able to propagate pro-inflammatory changes in naïve organotypic slice cultures (Crews et al., 2021). Moreover, they showed that depletion of microglia abrogates pro-inflammatory cargo of ethanol-induced EVs, suggesting that microglia-derived EVs may be a potential route of ethanol-induced neuroinflammation.

#### Main microglial pathways involved in alcohol use disorder: TLR4, NLRP3 inflammasome and complement system

At the mechanistic level, ethanol is thought to mediate its immune activation primarily via the PRRs-TLR4 and NLRP3, both of which are expressed predominantly in microglia in CNS (Zhang et al., 2014). TLR4, a member of the toll-like receptor family, is a transmembrane protein. Its activation leads to inflammatory cytokine production primarily through the NF-kB pathway (Kawasaki and Kawai, 2014). Increased TLR2, 3, 4 immunoreactivity has been reported in alcoholic brain tissue (Crews et al., 2013). Ethanol increases the expression of TLR2, 4 in microglia and astrocytes as well as increases their clustering in Caveolin positive lipid rafts (Blanco et al., 2008; Fernandez-Lizarbe et al., 2008, 2013). Deficiency in TLR4 or TLR2 provides protection against a broad range of pro-inflammatory changes mediated by alcohol (Table 2). Indeed, TLR4 or TLR2 deficiency attenuates ethanol-induced cytokine and chemokine release, activation of immune mediators (iNOS, COX-2), increased ROS, neuronal death, and anxiety like behavior (Fernandez-Lizarbe et al., 2009, 2013; Alfonso-Loeches et al., 2010; Pascual et al., 2015). In addition, TLR4 KO models provide protection against ethanol-induced microglia proliferation, infiltration of peripheral macrophages, activated microglia morphology and NLRP3 activation (Alfonso-Loeches et al., 2010, 2016). Ethanol treatment also induces TNFα responses in the cerebellum through induction of miR-155, in a TLR4-dependent manner (Lippai et al., 2013a).

Beyond TLR4, NLRP3 inflammasome is also responsible for ethanol-induced immune activation in the CNS (Table 2). Inflammasomes are cytosolic multiprotein complexes that regulate the release of cytokines (Broz and Dixit, 2016). Increased NLRP3 immunoreactivity and inflammasome activation was reported in post-mortem AUD brain samples, preclinical models of AUD and cultured microglia (Zou and Crews, 2012; Lippai et al., 2013b; Alfonso-Loeches et al., 2016). We have shown that genetic deficiency of NLRP3, the adaptor protein ASC, or inhibition of inflammasome signaling using IL-1Ra (anakinra) provides partial protection against ethanol mediated increases in TNFα, MCP-1, IL-1β (Lippai et al., 2013b). Moreover, inflammasome inhibition (NLRP3 inhibitor -MCC950 and Caspase-1 inhibitor- VX765) results in decreased alcohol preference in two-bottle choice test in mice (Lowe et al., 2020a). In hippocampal slice cultures, inhibition of inflammasome signaling by an IL-1β neutralizing antibody protects against ethanol-induced loss of neurogenesis (Zou and Crews, 2012).

The complement system mediates the recognition and elimination of pathogens and cellular debris by innate immune cells by guiding their phagocytic function. Seminal work by Stevens et al. and Paolicelli et al. shaped our understanding of the role of the complement system in microglia activity and synaptic pruning under homeostatic and disease conditions (Stevens et al., 2007; Paolicelli et al., 2011; Furman et al., 2012; Hong et al., 2016). In the CNS, complement proteins are mainly expressed in microglia and astrocytes (Zhang et al., 2014; Dalakas et al., 2020). Excessive alcohol consumption has been linked to increased expression of complement components *in vivo* in Raphe nucleus, cerebellum and hippocampus (Lowe et al., 2020b; Lee et al., 2021). Alcohol treatment of microglia cultures or organotypic slice cultures also shows increased expression of complement components, which can have neurotoxic effects (Kalinin et al., 2018; Mukherjee et al., 2020; Crews et al., 2021). However, there are contrasting reports of a reduction in *C1q* levels in frontal cortex of mice treated with ethanol (Socodato et al., 2020). Thus, further investigation using deficiency of complement components can help delineate the role of complement system in ethanol-induced neurotoxicity and behaviors.

#### Insights from microglia depletion experiments in alcohol use disorder

In addition to microglia, other immune cell types in the brain (astrocytes, oligodendrocyte precursor cells, and endothelial cells) also express PRRs and contribute to ethanol-induced neuroinflammation (see section “Main microglial pathways involved in AUD: TLR4, NLRP3 inflammasome and complement system”). Specific contribution of microglia in ethanol-induced inflammation has been highlighted in studies where microglia were depleted in AUD models. Depletion of microglia by CSF1R inhibitor PLX6522 reduces ethanol-induced inflammatory signatures (such as TNFα, CCL2), increases anti-inflammatory markers (IL-10 and IL-4), and reduces alcohol consumption (Crews et al., 2017; Warden et al., 2020). Moreover, inhibition of microglia activation protects against ethanol-induced aberrant synapse loss, anxiety and depressive behavior (Socodato et al., 2020; Warden et al., 2020; Lee et al., 2021). Microglia-specific transcriptomic changes upon alcohol exposure identified several genes involved in interferon, TGFβ, TLR signaling, and genes associated with alcohol consumption (McCarthy et al., 2018; Erickson et al., 2019a), providing further insights into microglia specific regulation in AUD. Detailed reviews on the role of microglia ablation in ethanol-induced behaviors have been previously published (Erickson et al., 2019b).

### Mechanisms of innate immune cell infiltration and communication into the brain parenchyma in alcohol use disorder

The peripheral innate immune system communicates with the CNS to elicit an inflammatory response (Dantzer et al., 2008; Bettcher et al., 2021). The communication between these two systems is established through trans-vascular mechanisms that involve passive or active transport of molecules and cells across the BBB (Varatharaj and Galea, 2017). In addition, the communication between the peripheral immune system and the brain may also involve the circumventricular organs, which are fenestrated and highly permeable regions of the brain (Profaci et al., 2020), that are in close contact with the big reservoir of immune cells located in the pia layer of the brain meninges (Alves de Lima et al., 2020).

Peripheral myeloid cells, including monocytes and neutrophils, have been reported to invade the CNS in pathological conditions (Ajami et al., 2018; Jordao et al., 2019; Cugurra et al., 2021) such as TBI and stroke (Herisson et al., 2018; Alam et al., 2020). Although it is likely that same mechanism of infiltration can occur in alcohol use disorders (AUD), clinical evidence that confirms this hypothesis is lacking. However, preclinical studies suggest that immune cell infiltration into the brain parenchyma is a driver of neuroinflammation and a relevant pathomechanism in AUD. Several studies have reported the presence of infiltrating monocytes in the brain parenchyma of rodents exposed to alcohol (Alfonso-Loeches et al., 2016; Hanslik and Ulland, 2020). Some of these studies linked this invasion to an alcohol-mediated disruption of the BBB (Haorah et al., 2005; Xu et al., 2019), although we have demonstrated that the recruitment of peripheral monocytes relies on a chemotactic mechanism involving the CCR2/CCR5 axis (Lowe et al., 2020b). Hence, the pharmacological blockage of this pathway reverts monocyte infiltration together with microglia activation and other neuroinflammatory outcomes (Haorah et al., 2005). Since the pharmacological intervention referenced specifically targets infiltration of myeloid cells in the brain, monocyte infiltration could be considered a driver of CNS inflammation in this model.

In addition, chronic alcohol abuse causes an increase of systemic inflammation directly affecting the stability of the BBB and increasing the influx of pro-inflammatory mediators into the brain parenchyma (Singh et al., 2007; Alikunju et al., 2011). The BBB consists of specialized endothelial cells lining the cerebral blood vessels, surrounded by pericytes and astrocytes which can sense peripheral inflammation and subsequently drive neuroinflammation in the brain parenchyma (Profaci et al., 2020). Hence, endothelial cells and pericytes appear to mediate the neuroinflammatory mechanisms induced by alcohol (Haorah et al., 2007; Singh et al., 2007; Floreani et al., 2010; Drieu et al., 2020). Similarly, the glymphatic system, whose function specifically depends on the BBB-associated astrocytes, is also impaired by high doses of alcohol (Lundgaard et al., 2018).

### Liver-brain axis in alcohol use disorder

Chronic alcohol abuse has a detrimental effect on the brain and the liver. The liver is the major organ involved in the metabolization of alcohol, although alcohol can also be metabolized by certain brain cell types (Jin et al., 2021). Thus, the function of the liver and the brain are very tightly regulated in situations of alcohol abuse, and patients with AUD often develop alcoholic liver disease (ALD), which can progress from steatosis to cirrhosis (Addolorato et al., 2016). During these processes, the liver loses its capacity to detoxify alcohol, subsequently increasing the production and release of hepatotoxins, toxic lipids, and cytokines into circulation with the development of systemic inflammation (Gao et al., 2011). The increase of unmetabolized alcohol, alcohol metabolites, interleukins, microRNAs and other liver-derived byproducts in the blood stream might induce neuroinflammation and brain damage (Szabo and Lippai, 2014). Indeed, preclinical evidence has highlighted the role of liver IL-17A in the induction of glia activation and brain damage. Specifically the pharmacological and genetic intervention to block peripheral IL-17A restored brain function and reduced voluntary alcohol consumption in an alcoholic liver disease model (Ma et al., 2016). In addition, preclinical and clinical studies demonstrate that lower hepatic synthesis of beta-hydroxybutyrate is linked to depression and white matter alterations in AUD (Leclercq et al., 2020).

Epidemiological data indicate that a diverse array of liver pathologies such as ASH, NASH, and viral steatohepatitis, are associated with cognitive and neuropsychiatric decline (Perry et al., 2008; de la Monte et al., 2009; Colognesi et al., 2020). In addition, recent studies have connected liver and brain abnormalities in AUD, suggesting that liver dysfunction predicts severity of executive impairment in AUD patients (Crespi et al., 2019; Lanquetin et al., 2021). Beyond epidemiological studies, preclinical evidence indicates that neuroinflammation (in particular, microglia activation) is implicated in cognitive decline during liver failure (Butterworth, 2011; Zemtsova et al., 2011; McMillin et al., 2016; Sun et al., 2019; Balzano et al., 2020; Hsu et al., 2021). Among all liver disorders, hepatic encephalopathy clearly exemplifies the influence of liver on brain homeostasis (Felipo, 2013). In this pathology, ammonia is believed to be the main liver byproduct that contributes to brain dysfunction. Indeed, several studies have demonstrated that hyperammonemia leads to glia activation (Zemtsova et al., 2011), brain swelling, and cognitive decline (Balzano et al., 2020). Additionally, we propose how other liver byproducts are likely to modulate neuroinflammation (Figure 2):

1. *Liver derived cytokines*. Considering the liver is one of the main sources of cytokines in the body (Robinson et al., 2016), it is plausible that liver cytokines reach the blood stream and have the capacity to affect other organs including the brain. Since cytokines are large hydrophilic molecules, they might penetrate the BBB encapsulated in exosomes or exert their effects through endothelial cells without crossing the BBB (Salvador et al., 2021).
2. *Liver derived micro-RNAs*. miRNAs are a class of non-coding RNAs with the capacity to regulate a broad array of biological functions from transcription to translation. These molecules can be transported in the blood stream via EVs (Mori et al., 2019) that can cross the BBB and protect miRNA from degradation (Alvarez-Erviti et al., 2011). Several studies demonstrated that alcohol alters the miRNA profile in the liver and the brain (Lim et al., 2021). In particular, miRNA-155 is upregulated in alcohol abuse and acts as a master regulator of inflammation in both organs. We previously reported that miR-155 mediates alcohol induced neuroinflammation and brain injury (Lippai et al., 2013a). Consequently, mice deficient in miR-155 were protected from pro-inflammatory cytokines and brain injury following ethanol exposure. Since the mice used in this study were constitutive knockouts (KO), the extent of contribution of liver miR-155 in the effects observed in the brain remains unknown.
3. *Liver exosomes and lipids*. As previously mentioned (see section “Release of extracellular vesicles by microglia”), exosomes produced in the liver and secreted into circulation can cross the BBB to reach the brain parenchyma, acting as a direct communicator between the liver and brain. In addition, bioactive lipid- ceramides are upregulated in alcoholic steatohepitits (Longato et al., 2012; Ramirez et al., 2013). Ceramides are important in exosome biogenesis and can cross BBB to exert neuroinflammatory effect (de la Monte et al., 2009).

## Brain innate immunity in Alzheimer’s disease

Alzheimer’s disease (AD) is a neurodegenerative disorder that manifests as memory loss and cognitive decline, and is characterized by accumulation of Aβ and the formation of intracellular neurofibrillary tangles (Heneka et al., 2015; Ennerfelt and Lukens, 2020). Recent genome wide association studies demonstrated the relevance of innate immunity in the pathogenesis of AD. These genetic findings are supported by preclinical and clinical research that explains the role of innate immunity in AD (Heneka et al., 2015; Ennerfelt and Lukens, 2020). In this section, we highlight mechanisms linked to AUD that are also involved in the progression of AD, and discuss AD targets that could be relevant to explore in AUD. We believe that convergent mechanisms may explain the plausible involvement of alcohol abuse in the progression of AD.

### Main microglial pathways involved in Alzheimer’s disease

#### Common players described in alcohol use disorder and Alzheimer’s disease: TLR4, NLRP3 inflammasome, and complement mediated mechanisms in microglia

In the CNS, TLR4 expression is enriched in microglia, and its activation is involved in the progression of AD and cognitive decline (Walter et al., 2007; Balducci et al., 2017; Zhou et al., 2020). Multiple lines of evidence highlight the involvement of TLR4 in the progression of AD and activation of these receptors was observed in microglia surrounding Aβ plaques (Walter et al., 2007), suggesting that Aβ peptides can be sensed through TLR4 (Walter et al., 2007). In addition, stimulation of TLR4 in microglia modulates the cell’s capacity to engulf Aβ plaques and subsequently affect disease progression (Wendeln et al., 2018).

While several inflammasomes are implicated in AD, the NLRP3 inflammasome in particular has been linked to the progression of AD (Dempsey et al., 2017; Heneka, 2017; Ising et al., 2019). Pharmacological intervention or genetic deletion of NLRP3 in preclinical models of familial AD, reduced Aβ plaques (Heneka et al., 2013), tau pathology (Ising et al., 2019), synaptic dysfunction and cognitive decline (Heneka et al., 2013). Furthermore, different studies have demonstrated that Aβ or tau aggregates activate the NLRP3 inflammasome, causing the subsequent release of IL-1β, Caspase-1 and ASC specs that perpetuate the aggregation of Aβ via a positive feedback loop (Venegas et al., 2017; Luciunaite et al., 2020).

As mentioned earlier (see section “Main microglial pathways involved in AUD: TLR4, NLRP3 inflammasome and complement system”) complement system plays essential role in microglia mediated phagocytosis during development and disease. This cascade is involved in AD, where Aβ mediated activation of C1q and C3 complexes guide microglia toward synapse pruning and elimination (Schafer et al., 2016; Wu et al., 2019). In the context of Aβ elimination, findings are controversial, and inhibition of this cascade has been linked with increased (Maier et al., 2008) and decreased amyloid load (Schafer et al., 2016; Wu et al., 2019).

#### Alzheimer’s disease-related mechanisms unexplored in alcoholism: disease associated microglia

Depending on their pattern of cytokine expression, microglia may be classified as M1 (inflammatory) or M2 (anti-inflammatory). This classification, although still used and useful for some approaches, is too simplistic to describe the complex physiology and phenotypes of these cells. The development of single cell approaches has allowed researchers to define cell populations of microglia associated with disease. Initially, disease associated microglia (DAM) was an example of a unique microglia population that evolves during the progression of Alzheimer’s pathology. This population is characterized by the loss of homeostatic function, with a pattern of expression that does not occur under the classical M1 or M2 phenotypes, and is conserved in mouse and humans (Butovsky et al., 2014; Keren-Shaul et al., 2017; Deczkowska et al., 2018). A similar microglia subset was found in ALS, FTD and aging, and DAM became a common feature among neurodegenerative diseases. This population is characterized by the expression of markers such as TREM2 or APOE, genes that were previously associated with genetic risk factors for AD and other neurodegenerative disorders, although the functional role of this cell subset is still unclear (Butovsky et al., 2014; Keren-Shaul et al., 2017; Deczkowska et al., 2018). In AD, DAM has been linked to the presence of amyloid plaques, but its association to neural cell death or synapse loss remains unknown. Since Aβ plaques are absent in other neurodegenerative pathologies in which DAM is present, common signaling patterns across neurodegenerative diseases that trigger the appearance of this subset is possible. Therefore, an open question remains whether similar populations exist in models of alcohol abuse, or whether alcohol influences the progression of DAM in neurodegenerative models. One feature that seems to be clear is that DAM appears in conditions in which there is accumulation of apoptotic neurons and myelin debris, and such features are present in the brain pathology associated with AUD (Vargas et al., 2014; Guo et al., 2021; Liu et al., 2021; Qin et al., 2021). Furthermore, multiple transcriptional studies reported that alcohol consumption modulates the expression of TREM2 in microglia cells (Erickson et al., 2019a), and TREM2 is required for DAM activation (Krasemann et al., 2017).

### Systemic inflammation and immune cell infiltration in Alzheimer’s disease

Multiple lines of evidence highlight the role of systemic inflammation in the progression of AD. Epidemiological studies consistently report that patients with AD have higher levels of proinflammatory cytokines (such as TNF and IL-6) (Kim et al., 2017; Bettcher et al., 2021) in their blood. In addition, genetic studies have provided evidence on the etiological role of innate immunity in AD (Lambert et al., 2013; Jansen et al., 2019; Wightman et al., 2021). These studies suggest that the potential causative genes for AD are strongly expressed in immune-related tissues (including liver and spleen) and in immune cell types (such as microglia cells within the CNS). Since then, microglia has been well studied, but the importance of peripheral innate immune cells inducing brain pathology cannot be ruled out. In support of this idea, recent studies highlighted the relevance of peripheral interventions that target innate immune cells as a strategy to revert neurodegeneration and cognitive decline (Bettcher et al., 2021).

Finally, monocytes and neutrophils are recruited to the AD brain, a process that happens as a result of a leaky BBB due to vascular injury (Sweeney et al., 2018), or following chemoattractant mechanisms such as the CCR2/CCL2 or CX3CR1/CX3CL1 axis in the case of monocytes (Naert and Rivest, 2011). Infiltration due to damage in the BBB is linked with neuroinflammation, microglia activation, and reduced synaptic plasticity (Fiala et al., 2002; Sweeney et al., 2018); infiltration that follows chemoattractant mechanisms is linked with neuroprotection. Hence, CCL2 positively recruited monocytes are better phagocytes than microglial cells with a higher capacity to engulf Aβ decreasing amyloid pathology and disease progression (Naert and Rivest, 2011; Guedes et al., 2018).

### Liver-brain axis in Alzheimer’s disease

The main lines of evidence linking the liver to AD involve the metabolism of lipids (Sutphen et al., 2015; Kim et al., 2016; Nho et al., 2019; Khodabakhsh et al., 2021) and bile acids (Sutphen et al., 2015; Nho et al., 2019), and the role of the liver in the clearance of circulating Aβ (Estrada et al., 2019). As previously mentioned, the liver is in charge of the metabolic detoxification and clearance of the bloodstream to avoid multi-organ failure. In the context of AD, the process of metabolic clearance involves the degradation of systemic Aβ loading. Bile acids and hepatocytes mediate the degradation and uptake of circulatory Aβ, respectively. Specifically, LRP-1 expressed in hepatocytes, binds to Aβ and mediates its uptake from the circulation. The expression of LRP-1 is drastically reduced in conditions of alcohol abuse, obesity or diabetes (Sutphen et al., 2015; Xiang et al., 2015; Estrada et al., 2019), which might subsequently affect the clearance of Aβ.

Furthermore, brain features of AD including accumulation of aberrant lipids and increased abundance of Aβ were observed in a transgenic mouse model in which the synthesis of human amyloid was restricted to the liver (Lam et al., 2021). These mice presented clear signs of neuroinflammation and neurodegeneration together with cognitive decline. This study demonstrates the importance of the liver in maintaining brain homeostasis in the context of AD, and serves as proof that the toxicity of the periphery, specially coming from the liver, can affect the CNS.

In addition, liver deterioration induced by high sugar and high cholesterol diets accelerates AD pathology and cognitive decline (Kim et al., 2016), providing further evidence of the involvement of liver failure in the deterioration of brain function.

Beyond Aβ clearance, the liver is involved in the production and metabolism of lipids. Lipid metabolism, together with neuroinflammation, is one of the main pathomechanisms involved in the pathology of AD (Jansen et al., 2019; Chew et al., 2020), although it has been primarily studied in the context of the CNS. Clinical and preclinical studies have recently highlighted that the metabolism of lipids is impaired at systemic level and not just in the CNS of AD patients, and even peripheral lipid metabolites were proposed as candidate biomarkers to predict cognitive decline in AD (Beal, 2011). Although it is still not clear how the metabolism of lipids in the periphery affect the CNS, we cannot exclude the hypothesis that these lipids could communicate across the BBB (Andreone et al., 2017; Pifferi et al., 2021) or even wrongly influence the reprogramming of innate immune cells (Batista-Gonzalez et al., 2019) (Figure 2).

## Alcohol abuse and progression of Alzheimer’s disease

### Clinical studies

Many studies have evaluated the effect of alcohol abuse on brain function, dementia and cognitive decline. However, the field lacks epidemiological data investigating the effect of alcohol in AD. Thus, as far as we know, the available data do not support a strong association between alcohol consumption and AD (Tyas, 2001), although some studies suggest a positive association (Schwarzinger et al., 2018).

Despite inconclusive responses from epidemiological analyses, genetic studies indicate that light or moderate consumption of alcohol later in life is associated with learning and memory deficits in AD patients that carried an *APOE 4* mutation, as APOE was highly enriched in astrocytes and microglia cells in the CNS (Koch et al., 2019). In addition, a recent study showed overlapping genetic risk factors (such as *SPI1* or *MAPT*) in AD and AUD (Kapoor et al., 2021). This study combined genetic, transcriptomic and epigenetic data to support molecular commonalities among these disorders, highlighting the role of the innate immunity.

Beyond AD, the association of alcohol abuse with other forms of dementia such as alcohol related dementia, Korsakoff Syndrome, or vascular associated dementia is more clear (Schwarzinger et al., 2018), and the role of alcohol in the brain pathology of these syndromes has been previously reviewed (Wiegmann et al., 2020; Parial et al., 2021; Visontay et al., 2021).

### Preclinical studies

Dementia and neurodegeneration, the hallmarks of AD, are also observed in AUD patients and preclinical models (Obernier et al., 2002; Alfonso-Loeches et al., 2010; Qin and Crews, 2012b; Coleman et al., 2017; Lundgaard et al., 2018; Mira et al., 2019). However, studies evaluating the direct effect of alcohol on Aβ plaques or neurofibrillary tangles are limited. Chronic alcohol administration exacerbated memory deficits and sensorimotor processing in the 3X-Tg mouse model of AD (Hoffman et al., 2019). In this model alcohol caused up-regulation of Aβ and hyperphosphorylated tau in a region-specific manner (Ibanez et al., 2019). Similarly, four weeks drinking in the dark paradigm exacerbated memory deficits and Aβ plaques in APP23/PS45 mice. The authors observed that ethanol treatment increased APP and BACE1 levels *in vitro* neuronal cell line and in the AD mouse model. Interestingly, a model of moderate alcohol consumption, was found to improve memory performance of 3X-Tg mice, possibly through the effect of alcohol on Aβ aggregation (Belmadani et al., 2004). Although these studies suggest that heavy alcohol consumption may exacerbate AD symptoms, the causative role of innate immune activation in the CNS and periphery in this process remains unknown. Primary microglia cultures treated with ethanol show reduced engulfment of Aβ, suggesting a possible link between increased Aβ burden and alcohol abuse (Kalinin et al., 2018). Considering the commonalities in the innate immune activation in AD and AUD, it is tempting to speculate that alcohol induced-innate immune reactions in the CNS and periphery will exacerbate AD pathology, although a direct relationship remains to be established (Crews, 2008). Moreover, a detailed investigation into the dose of alcohol and the stage of progression of AD needs to be considered while evaluating the effect of alcohol-induced inflammation in AD.

## Conclusion

As reviewed here, compelling evidence suggests that AD and AUD share common pathological mechanisms that involve the activation of the innate immune system. In both disorders there is clear evidence for the role of microglia, and both disorders can be viewed as systemic diseases in which the peripheral compartments and liver can regulate and influence the response of the CNS. Despite initial preclinical and clinical evidence of these shared pathomechanisms, the specific role of alcohol abuse in AD progression has not been disentangled and larger longitudinal epidemiological studies and preclinical research in AD models in the context of alcohol abuse is awaited.

## Author contributions

AR, RJ, and GS wrote and edited the manuscript. All authors contributed to the article and approved the submitted version.
